# Direct interaction of metastasis-inducing S100P protein with tubulin causes enhanced cell migration without changes in cell adhesion

**DOI:** 10.1042/BCJ20190644

**Published:** 2020-03-27

**Authors:** Min Du, Guozheng Wang, Igor L. Barsukov, Stephane R. Gross, Richard Smith, Philip S. Rudland

**Affiliations:** 1 Institute of Integrative Biology, University of Liverpool, Crown Street, Liverpool L69 7ZB, U.K.; 2 Institute of Infection and Global Health, University of Liverpool, Crown Street, Liverpool L69 7ZB, U.K.; 3 School of Life and Health Sciences, Aston University, Aston Triangle, Birmingham B4 7ET, U.K.

**Keywords:** cell migration, metastasis, microtubule, peptides, S100P, tubulin

## Abstract

Overexpression of S100P promotes breast cancer metastasis in animals and elevated levels in primary breast cancers are associated with poor patient outcomes. S100P can differentially interact with nonmuscle myosin (NM) isoforms (IIA > IIC > IIB) leading to the redistribution of actomyosin filaments to enhance cell migration. Using COS-7 cells which do not naturally express NMIIA, S100P is now shown to interact directly with α,β-tubulin *in vitro* and *in vivo* with an equilibrium *K*_d_ of 2–3 × 10^−7^ M. The overexpressed S100P is located mainly in nuclei and microtubule organising centres (MTOC) and it significantly reduces their number, slows down tubulin polymerisation and enhances cell migration in S100P-induced COS-7 or HeLa cells. It fails, however, to significantly reduce cell adhesion, in contrast with NMIIA-containing S100P-inducible HeLa cells. When taxol is used to stabilise MTs or colchicine to dissociate MTs, S100P's stimulation of migration is abolished. Affinity-chromatography of tryptic digests of α and β-tubulin on S100P-bound beads identifies multiple S100P-binding sites consistent with S100P binding to all four half molecules in gel-overlay assays. When screened by NMR and ITC for interacting with S100P, four chemically synthesised peptides show interactions with low micromolar dissociation constants. The two highest affinity peptides significantly inhibit binding of S100P to α,β-tubulin and, when tagged for cellular entry, also inhibit S100P-induced reduction in tubulin polymerisation and S100P-enhancement of COS-7 or HeLa cell migration. A third peptide incapable of interacting with S100P also fails in this respect. Thus S100P can interact directly with two different cytoskeletal filaments to independently enhance cell migration, the most important step in the metastatic cascade.

## Introduction

The major cause of death from the commonly occurring cancers is due to metastasis, the process of tumour dissemination from its primary site to distant organs [1]. This dissemination process is the major obstacle to successful treatment of breast and other commonly occurring cancers, since the primary tumour can be surgically excised successfully, but the disseminated disease is much more difficult to treat [2]. Metastasis occurs in multiple steps, the first and perhaps the most important step is the cancer cells’ ability to migrate away from the primary tumour [3]. Although multiple steps are required for the metastatic process, certain gene products, called metastasis-inducing proteins [4] can induce metastasis when expressed in rodents [5], providing they can act on a cell which is already neoplastic [6]. One such group includes some members of the S100 family of small, calcium-binding regulatory proteins [5]; one of the best studied is S100P [7,8]. Our previous work demonstrated that S100P promotes cell migration and induces metastasis in an animal model of breast cancer, and that elevated levels of S100P in primary human breast cancer are associated with shorter patient survival times [9]. More recently, we found that S100P differentially interacts with nonmuscle myosin (NM) isoforms *in vitro* and *in vivo*, binding preferentially to NMIIA rather than to NMIIB or IIC. This differential interaction causes the redistribution of the microfilaments and focal adhesion sites and thereby facilitates, in part, increases in migration of the S100P-overexpressing cells [10]. This work, together with that on S100A4 [11,12] demonstrates that the effect of both these S100 proteins on the actomyosin cytoskeleton is important in cancer cell migration *in vitro* and on metastasis *in vivo*. This interaction between the cytoskeleton and cell motility can also occur in noncancerous cells such as trophoblasts [13].

The cytoskeleton, however, is composed of three types of filaments, microfilaments containing nonmuscle actin and myosin, microtubules (MTs) containing α, β-tubulins, and intermediate filaments containing cytokeratins/vimentin. The first two types of filaments are both important in cell migration [14]. Nonmuscle actomyosin filaments are the major machinery of cell adhesion, polarity and migration, whilst MTs are active components of the motile machinery of the reticulopodia [15], flagella and the tails of sperm [16]. However, in contrast with the actomyosin filaments, there are few definitive experiments to show that MTs are actively involved in cancer cell migration. Stabilisation of MT by chemicals, such as taxol, normally inhibits cell migration [17,18], whilst the effects of MT-disrupting reagents, such as colchicine and nocodazole, are dependent on the cell type [19,20]. The cellular response to such MT-disrupting agents in the short term is to increase cell migration in some cells, but to decrease it in others [21]. Theoretically, the MTs radiating from the MT organising centres (MTOCs) to the cellular peripheries provide strong resistance to changes in cell shape and to migration, unless the MTs are dynamically regulated or released from the MTOCs [22].

The individual S100 proteins have been reported to interact *inter alia* with either NM or with tubulin, although with different binding affinities [23]. For example, S100A1 and S100B can interact with tubulin in a calcium-dependent manner [24] and disrupt the assembly of MT [25]. Decreasing S100A1 is able to increase tubulin levels and this change alters neurite organisation in P12 cells [26]. In contrast, the S100A9/A8 (MRP14/MRP8) complex promotes tubulin polymerisation *in vivo* and is an essential component in the MAP-kinase-p38-induced migration of macrophages [27]. However, it is not known whether any member such as S100P can bind directly to tubulin as well as to NMIIA, and thereby also influence cell migration. To test this hypothesis we have established S100P-inducible cell lines from COS-7 cells which have been reported to contain no NMIIA [28] and studied the effects of S100P on cell migration and the possible involvement of the microtubular machinery in this NMIIA-deficient as well as in an NMIIA-intact cell system.

## Materials and methods

### Cell lines and cell migration assay

Rama 37 and S100P-inducible Rama 37, HeLa and S100P-inducible HeLa cells were produced and cultured as described previously [28]. The COS-7 monkey kidney cell line was obtained from ATCC cell bank and was reported to contain no NMIIA [28]. S100P-inducible COS-7 cells were generated as before for HeLa cells [10] with 2 plasmids: pBTE to express the doxycycline regulatory element rHTA2(s)-m2 and pTRE-ins to express the target protein [29]. Three inducible clones derived from the COS-7 cells were termed COS-7 S7, S10 and S23. The concentration of doxycycline and the incubation period were optimised at 1µg/ml and 24h for all the inductions and the level remained constant for at least a further 48 h. All cell lines are clonal in origin and hence have the same genetic background. They are used within five passages or about 15 generations to reduce variability due to spontaneous transformations.

Cell migration assays were performed using Boyden chamber transwells separated by a membrane with 8µm diameter pores, as described previously [10]. Usually, the cells were plated in Transwells and separately in 24 well plates without or with 1 µg/ml doxycycline and experiments terminated after 24 h incubation. The upper side of the membrane in the transwells was wiped clean, the lower side stained with Quick-Diff Kit (Polysciences, Germany) and the number of migrating cells counted (M). The total numbers of cells in the wells for growth control (G) were also counted using a cell counter. Cell migration rate (%) = M/G × 100. In order to standardise results between different experiments, the cell line controls were usually set to 100% migration and changes relative to their value were shown for most results. To test the effects of tubulin peptides on S100P-enhanced cell migration, 20 µM peptides tagged for cell entry were added 8 h after doxycycline-induction of S100P-inducible COS-7 cells in the Transwells, remaining procedures were the same as above. There was no discernable reduction in cell numbers on the membranes of parallel wells to those measuring transmembrane migration due to possible toxicity of tagged peptides, nor increase due to overexpression of S100P in agreement with a previous report [10].

### Cell adhesion assays

For kinetics of attachment either HeLa-A3 or COS-7 S10 cells were induced with 1 µg/ml doxycycline 48 h prior to experiment and 2 × 10^5^ uninduced or induced cells in 1 ml were added to 24 well tissue culture dishes and allowed to adhere at 37°C. At the given time (30 min to 2 h) cells were washed 2× with PBS and detached with 250 µl 0.05% (w/v) trypsin in versene for 5 min at 37°C, neutralised in serum-containing media (SCM) [10], counted and the percent of seeded cells recorded. For strength of adhesion, the same cells were seeded in 24 well tissue culture dishes so as to yield 80% confluence in 24 h. In all cases identical dishes were set up for test and control. After 24 h, wells were washed 2× and detached with 250 µl, 0.0125% (w/v) trypsin in versene for 5 min at 37°C. Wells were then carefully washed 2× to remove weak binders. Trypsin in versene (250 µl of 0.05% (w/v)) was then added to both test dish and control dish for 5 min to remove all cells from the well, neutralised with SCM and counted. The proportion of cells remaining after the weak digestion step was determined from the ratio of results from test and control plates. All assays were performed in triplicate.

### Production of tubulin/fragments

The coding sequences for full length (human) α-tubulin and for N-terminal and C-terminal halves of α or β-tubulin were generated using PCR with specific primers shown in Supplementary Table S1 and sub-cloned into pET16(b) for expressing and purifying His-tagged full-length α-tubulin and His-tagged tubulin fragments in *E. coli*, as described previously [10]. β-tubulin was purchased from Abcam.

### Gel overlay assay

Equal molar proteins (1 μM) were subjected to SDS PAGE on 10% (w/v) polyacrylamide gels. One gel was stained with Coomassie Blue to check equal loading and the other electroblotted onto PVDF membrane. Three µg/ml S100P in Gel Overlay Buffer were added to the membrane and the contents incubated at 4°C overnight. After extensive washing, the presence of bound S100P was probed with mouse anti-S100P as described for Western blotting.

### Western blotting

20 µg cell lysates were subjected to SDS–PAGE and electrically blotted on to PVDF membrane. After blocking with 2% (w/v) BSA, the blot was probed with rabbit anti-β-tubulin (Abcam), rabbit anti-actin (Abcam), rabbit anti-NMIIA (Abcam) or rabbit anti-S100P (R&D Systems). After extensive washing, anti-rabbit-HRP (Santa Cruz) 1/10,000 was used and the bands were visualised using ECL and their densities were measured using Image Lab software.

### Binding studies

For Western blotting, S100P was detected using mouse anti-S100P monoclonal antibody (mAb) (1 : 50 dilution) (BD Biosciences) which showed no cross-reaction with S100A1, A2 or A4 proteins [9]. Anti-tubulin (α, β), anti-actin from Sigma and anti-nonmuscle myosin IIA (NMIIA) from Covance (Princetown, NJ) were used as described previously [9,10]. α-tubulin was synthesised by recombinant means (Supplementary Materials and Methods) (Supplementary Table S1) and β-tubulin purchased from Creative BioMart (Shirley, U.S.A.).

For pulldown assays, S100P-inducible COS-7 cells were incubated with doxycycline for 16 h and lysed in 20 mM Tris–HCl pH 8.0, 150 mM NaCl and 1% (v/v) Nonidet P-40 (NP-40) with 0.5 mM CaCl_2_ or 1.0 mM EGTANa_2_. After brief sonication, the lysates were centrifuged at 15,000***g*** for 30 min to remove insoluble components. The supernatants were then applied to His-S100P columns or control column with His-binding beads only. After extensive washings with 10 mM Tris–HCl, pH 7.4, 150 mM NaCl, 0.5% (v/v) Tween 20, the bound proteins were eluted with 300 mM imidazole and then subjected to Western blotting using mouse anti-S100P mAb and anti-tubulin antibodies (R&D Systems, Abingdon, U.K.).

For co-immunoprecipitations, S100P-inducible COS-7 cells were incubated with 1 µg/ml doxycycline for 16 h and lysed in RIPA buffer (Thermo Fisher Scientific, Loughborough, U.K.) with 0.5 mM CaCl_2_. After sonication and centrifugation as described above, control antibody (rabbit IgG from Sigma), anti-tubulin or anti-S100P were added to three separate tubes containing 0.7 ml each of cell lysate and incubated overnight at 4°C. Then 100 µl Dynabeads® ProteinG (Thermo Fisher Scientific) were added to each tube and the contents incubated for 4 h at 4°C. After extensive washings with washing buffer (10 mM Tris–HCl, pH 7.4, 150 mM NaCl, 1% (v/v) Triton X-100, 0.2 mM sodium orthovanadate), the bound proteins were eluted with 0.1 M glycine pH 2.5 and then subjected to Western blotting using the paired antibodies together with a sample of the input and the last wash.

For SPR binding assays, α,β-tubulin proteins described earlier were immobilised on a carboxymethylated dextran surface of a CM5 chip using BS^3^ for use in Biocore X100. Recombinant S100P produced as described previously [10] was used as a ligand in different concentrations at 20, 50, 100, 200, 1000, 2000 nM for calculation of the equilibrium dissociation constant (*K*_d_) from the association/dissociation rate constants and from the extent of binding near equilibrium (Supplementary Table S2), as described previously [10,30]. To detect the effects of tubulin peptides on the interaction of tubulin, different concentrations of tagged and untagged peptides were mixed with 1 µM S100P first and the mixtures were then applied to the tubulin-coated chip. The relative changes in kinetics and binding extents with different peptides were calculated using software provided by the manufacturer.

### Immunofluorescent staining and confocal microscopy

Immunofluorescent staining was carried out as previously described [31]. COS-7 S10 and MCF-7 cells were induced for 48 h prior to plating at 25,000 cells/well onto either untreated or fibronectin-coated (2.5 µg/cm^2^) glass coverslips in a 24-well plate. After a further 48 h, cells were washed once in Cytoskeleton Buffer (CB: 150 mM NaCl, 5 mM MgC1_2_, 5 mM EGTA, 5 mM glucose, 10 mM 2-(N-morpholino) ethanesulfonic acid, pH 6.1) prior to fixing with 3.7% (w/v) paraformaldehyde in CB at 37°C for 20 min. Cells were permeabilised with 1% (v/v) Triton X-100 in CB for 2 min and blocked with 5% (v/v) goat serum in CB for 60 min before incubation with rabbit anti-EB3 (Abcam ab157217) and mouse anti-α-tubulin (Sigma T5168) (dilution between 1/400 and 1/500) in 1% (v/v) goat serum in CB for 60 min at room temperature. After extensive washing, cells were incubated with the appropriate secondary antibodies: anti-rabbit FITC (Dako F0205) and anti-mouse Alexa Fluor (Molecular Probes A11004), respectively, for 60 min at room temperature. After washing, coverslips were mounted on slides in Vectashield (Vector Labs, U.K.), before being viewed in an Epifluorescence Leica DMI400B microscope equipped with objective lens 63× Leica PH3 FLUOTAR 25 Oil and excitation at 488 nm, emission at 520 nm for FITC and excitation at 540 nm and emission at 570 nm for TRITC. Images were obtained using a Leica DFC360FX digital camera and the Leica LAS AF 6000 software. FITC-labelled antibodies to EB3 were used for EB3 staining. Quantification of the presence of EB3 clusters at the cell periphery or well-defined MTOC was performed by counting the number per cell in about 100 cells selected at random. Results are presented as percentages of the cell population containing either an EB3 network at the cell periphery or well-defined MTOCs. For MTOC quantification, cells and MTOC were graded as shown in Figure 4C′ where organisation is defined as (1) not visible or poorly structured organisation; (2) visible but poorly formed; (3) visible and defined; and (4) well defined and extensive regions. Cells showing 1–2 grading would be considered as poorly defined, whilst cells in 3–4 would be deemed as well structured. Scoring was undertaken by observers blinded to the identity of samples.

### Living colour expression and confocal microscopy

S100P fused to yellow fluorescent protein (YFP), and EB3 fused to cyan fluorescent protein (CFP) (EB3-CFP) expression plasmids, pYFP-S100P and pCFP-EB3 were constructed, respectively. COS-7 cells were transiently transfected with the two plasmids and images were recorded using a LSM 5 confocal microscope 24 h after transfection. CFP: excitation = 436 nm, emission = 480 nm; YFP: excitation = 500 nm, emission = 520 nm.

### In vivo tubulin polymerisation assay

HeLa and S100P-inducible HeLa, COS-7 and S100P-inducible COS-7 cells were routinely cultured with or without doxycycline (1 µg/ml) and treated with 33 µM nocodazole for 24 h to synchronise the cells. Then the nocodazole was washed away (First release) and the synchronised cells were cultured in normal medium for 4 h to allow them to complete cell division. The nocodazole was then added again for 4 h to dissociate all the MTs. After this time, nocodazole was again removed by extensive washings (Second release) and tubulin was allowed to polymerise in the interphase of the cell cycle for different times [32,33]. The cells were treated with buffer (10 mM Tris–HCl, 150 mM KCl, pH 7.4, 2% (v/v) Triton X-100) and the Triton-X100-insoluble tubulins (representing the newly formed MTs) were solubilised in Sample Buffer and quantified by Western blotting. To test the effect of tubulin-derived peptides, 20 µM T-DIC, T-DLV, T-IMN and tag-peptide alone (Supplementary Table S3) in 0.25% (v/v) DMSO as solvent, as well as the solvent control 0.25% (v/v) DMSO were added immediately after the second release in Sample Buffer and quantified at the different time points by Western blotting.

### Trypsin digestion, affinity chromatography, and mass spectrometry

Fifty micrograms of α and separately β-tubulins were incubated with 5 µg trypsin in 200 μl total volume at 37°C overnight in 25 mM ammonium bicarbonate to ensure complete digestion. Resultant peptides were diluted in 10 ml Binding Buffer (20 mM Tris–HCl pH 8.0, 150 mM NaCl, 0.5 mM CaCl_2_ and 0.5% (v/v) Tween 20) and applied to a S100P-conjugated CNBr-Sepharose 4B column in the presence of 0.5 mM CaCl_2_. After extensive washings, the bound peptides were eluted with 0.1 M glycine pH 2.5 and neutralised immediately. These eluates were analysed using the mass spectrometry service provided by the University of Dundee using nanoLC–MS–MS. The raw data was analysed further using Peak software 7.0 and all identified peptides were superimposed on the amino acid sequence of α or β-tubulin.

### Peptide synthesis

Mass spectrometry-identified tubulin peptides were first analysed for their appearance on the surface of the 3D structure of α or β tubulin [34] using PyMol software (Schrödinger, Inc.). Five peptides of 25–30 amino acids long which mapped to the tubulins’ surface were chosen and synthesised commercially by Syn Peptides (Shanghai, China) to 98% purity. For *in vivo* experiments, the 10 amino acid TAT-HA2 tag for cell entry [35] was added to selected peptides and new peptides with entry tags were synthesised by the same company (Supplementary Table S3).

### NMR spectroscopy

^1^H, ^15^N HSQC spectra were measured at 40°C in 20 mM 2-morpholinoethanesulfonic acid sodium salt (MES), 50 mM NaCl, 0.5 mM CaCl_2_, 2 mM Tris-carboxyethyl-phosphine (TCEP), pH 6.1 buffer as described previously [36]. Samples were prepared from 1.6 mM solution of ^15^N-labelled S100P and 2–5 mM peptide solutions to obtain a 1 : 1 mixture of 0.1 mM of S100P and 0.1 mM of each peptide. Initially, peptide solutions were prepared by weight to produce 5 mM peptide concentrations. Some peptides had low solubility and did not fully dissolve, leading to visibly cloudy solutions. In these cases, buffer was added to reduce the peptide concentration to 2 mM. In the case of two peptides, DLV and VNA, solutions remained cloudy. These peptides were added to the protein as cloudy suspensions to produce 0.1 mM solutions; some precipitations were still detected. To estimate the peptide concentrations, equivalent peptide solutions without the protein were prepared. All solutions were centrifuged before the NMR measurements. NMR spectra were collected on Bruker Avance III 800 MHz spectrometer equipped with CryoProbe. Data were processed and analysed in TopSpin 3.5 (Bruker, Karlsruhe, Germany).

### Isothermal titration calorimetry (ITC)

Measurements were conducted using an ITC-200 instrument (Microcal) as previously described [36]. ITC titrations were performed in 20 mM MES pH 6.1, 50 mM NaCl, 1.0 mM TCEP and 5 mM CaCl_2_ (for Ca^2+^-form of S100P) at 25°C. Due to low solubility, peptide solutions at concentrations of 10–400 µM were placed in the cell and titrated with S100P at concentrations of 0.5–3 mM, depending on the peptide concentration. Data were integrated and fitted to a single site binding equation using Origin 7 software which was included with the ITC module (Malvern Microcal, Malvern, U.K.).

## Results

### S100P overexpression increases cell migration but does not reduce cell adhesion in myosin IIA-deficient COS-7 cells

**Figure 1.**
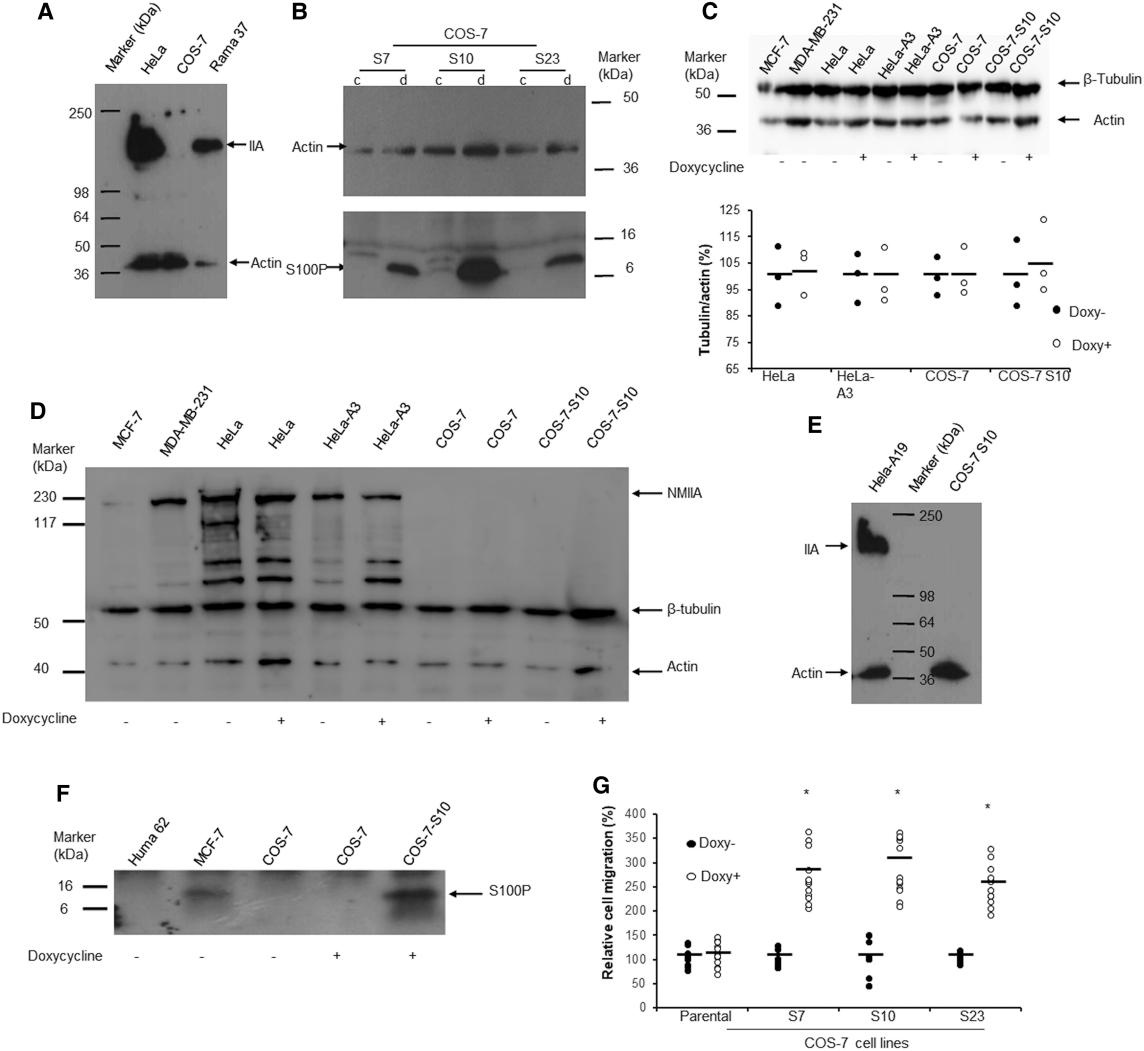
S100P enhances cell migration in COS-7 cells with undetectable nonmuscle myosin IIA. (**A**) Western blot with anti-nonmuscle myosin IIA and anti-β-actin. There was no band at 226 kDa corresponding to nonmuscle myosin IIA (IIA) in monkey COS-7 cells compared to that seen in human HeLa and rat Rama 37 cells. The gels have been overexposed to detect even the smallest trace of NMIIA. (**B**) Three clones of S100P-inducible COS-7 cells (S7, S10, S23) were exposed to 0 (c) or 1 µg/ml (d) doxycycline for 24 h. Western blot of equal amounts of loaded proteins shows relative S100P levels. In addition to monomeric S100P at an apparent mol wt of 10 kDa (arrow), multimeric forms can also be seen in these overexposed gels. (**C**) Effects of doxycycline on the abundance of tubulin. Upper panel: Typical Western blot for β-tubulin and actin in cell lines treated without (Doxy−) or with (Doxy+) 1 µg/ml doxycycline for 24 h using both rabbit anti-β-tubulin (Abcam) and rabbit anti-actin (Abcam) (Methods). Lower panel: the relative percentage (%) ratios of band densitites of tubulin/actin in dot plots with bars representing means showed no significant difference between paired doxycycline-treated cells and control cells (Students *t*-test, *P* > 0.05). (**D**) NMIIA abundance in cell lines. Western blot was performed simultaneously using rabbit anti-nonmuscle myosin IIA (NMIIA) (Abcam), rabbit anti-β-tubulin (Abcam) and rabbit anti-actin (Abcam) to detect protein levels of NMIIA, β-tubulin and actin in cell lines as indicated from cultures treated without (−) or with (+) 1 µg/ml doxycycline for 24 h (Methods). No NMIIA was detected in the COS-7 cell lines under either condition. MCF-7 produced NMIIA, but at a lower level than MDA-MB-231 and HeLa cell lines. Extra, perhaps nonspecific protein bands were seen in HeLa cells with this anti-NMIIA antibody. (**E**) Western blot for NMIIA (IIA) and actin in S100P-inducible HeLa-A19 and COS-7 S7 S10 cells treated for 24 h with 1 µg/ml doxycycline. There was no band corresponding to NMIIA in treated or untreated COS-7 cells S10 cells. (**F**) S100P abundance in cell lines. Western blot was performed using anti-S100P antibody (R&D Systems) 1/1000 on cell lysates from cultures of Huma 62 (Human Breast benign cell line for negative control), MCF-7, COS-7 parental cells without (−) or with (+) 1 µg/ml doxycycline for 24 h and COS-7-S10 (S100P-inducible) cells with (+) doxycycline under the same conditions (Methods). S100P protein was detected only in MCF-7 and COS-7-S10 treated with doxycycline. (**G**) Cell migration assay of parental COS-7 (set at 100%) and COS-7 S7, S10 and S23 cells in the absence (Doxy−) or presence (Doxy+) of 1 µg/ml doxycycline for 24 h in dot plots with bars representing means. *Student's *t*-test, *P* < 0.01 when compared with non-induced control.

In our previous study, we reported that S100P enhances cell migration by binding to and affecting distribution of NMIIA filaments in HeLa and Rama 37 cells [10]. We now sought to determine whether S100P overexpression could induce such changes in cells when NMIIA was absent. The monkey kidney COS-7 cells have been reported to lack NMIIA [28] and this was confirmed in comparison with its presence in human HeLa and rat Rama 37 cells (Figure 1A). We then generated three S100P-inducible COS-7 cell lines using doxycycline as the inducing agent, the largest increase being in clone S10 (Figure 1B). When 1 µg/ml doxycycline was added to COS-7 S10 cells, the increase in S100P peaked at 24 h and remained constant for at least 72 h without effect on the expression of tubulin and non-expression of NMIIA (Figure 1C,D). This clone still contained no NMIIA whilst the S100P-inducible HeLa cells, HeLa-A19 still expressed NMIIA upon S100P induction (Figure 1E). There was no change in levels of tubulin, no detectable NMIIA nor any S100P when the same concentration of doxycycline was added to parental COS-7 cells (Figure 1C,D,F). When doxycycline was added for 24 h to the three inducible COS-7 cell lines, the relative cell migration rates were also significantly increased by 2.5 to 2.9 fold but that for parental COS-7 cells, was not (Figure 1G). In comparison overexpression of S100P in the NMIIA-containing inducible Rama 37 and HeLa cells caused a 3.2 to 4.8 fold increase in migration rates [10,37]. In contrast with the S100P-inducible cell line HeLa-A3 (Student's *t*-test *P* ≤ 0.022), there was no significant decrease in the rate of adhesion (Supplementary Figure S1) or significant decrease in strength of adhesion (Supplementary Figure S2) of S100P-inducible COS-7 S10 cells (*P* ≥ 0.18) upon addition of 1 µg/ml of doxycyclin. These results indicate that S100P can enhance cell migration through mechanisms other than via NMIIA and cell adhesion in the COS-7 cells.

### S100P interacts with tubulin *in vitro*

**Figure 2.**
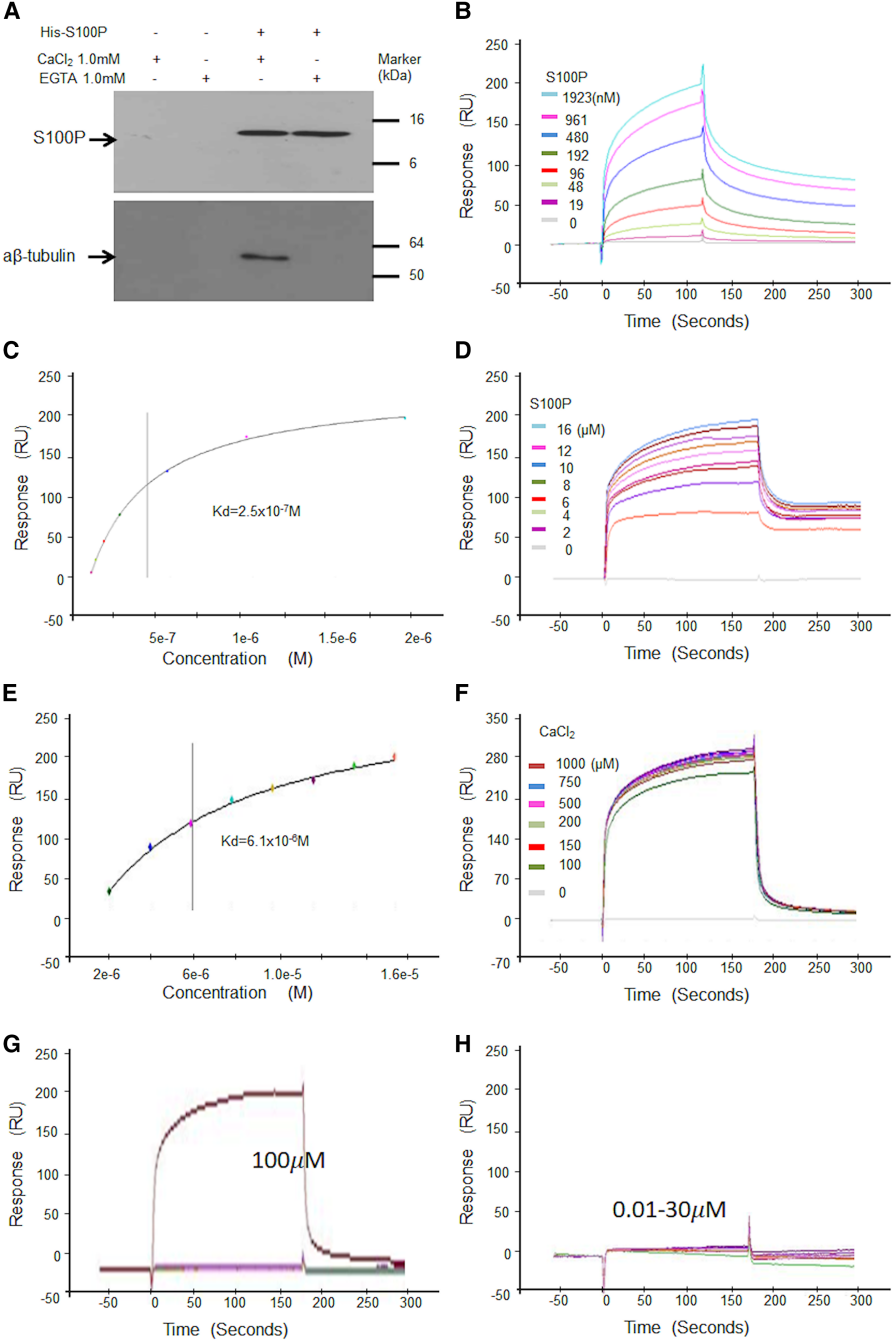
S100P binds to α,β-tubulins *in vitro*. (**A**) Pulldown assay. His-S100P was loaded on His-binding beads. COS-7 cell lysates were applied to control beads (His-binding beads only) (a and b) or His-S100P beads (c and d) in the presence of 1 mM CaCl_2_ (a and c) or EGTA (b and d). After extensive washings, bound proteins were eluted with 300 mM imidazole from each bead and any His-S100P or α, β-tubulins were detected by Western blotting using anti-S100P (Upper panel) or anti-tubulin (Lower panel) antibodies. (**B**–**F**) Surface Plasmon Resonance (SPR) binding assays using α,β-tubulin immobilised on a chip and different concentrations of S100P in solution. (**B**) Typical binding curves of response units (RU) in arc seconds against time in physiological buffer of 0.15 M NaCl with 200 µM CaCl_2_ and multiple cycles of different concentrations of S100P. (**C**) A typical corresponding graph of response (RU) plotted against semilog of different concentrations of S100P for calculation of dissociation constant (*K*_d_) at or near equilibrium for one experiment. (**D**) Typical binding curves of response (RU) in arc seconds in high salt buffer (0.5 M NaCl) with 200 µM CaCl_2_ and multiple cycles of different concentrations of S100P. (**E**) Corresponding graph of response (RU) plotted against semilog of different concentrations of S100P for calculation of *K*_d_ at or near equilibrium for one experiment. (**F**) Typical binding curves in physiological buffer of 0.15 M NaCl for fixed concentration of 2 µM S100P and multiple cycles of different concentrations of CaCl_2_. The values of the means ± SE from three experiments are quoted in Supplementary Table S2 (**G** and **H**). Typical binding curves of S100P (2 µM) to immobilised tubulin showing response units (RU) in arc seconds plotted against time for lower concentrations of CaCl_2_ in physiological buffer containing 0.15 M NaCl. The different concentrations of CaCl_2_ used were 0, 0.01, 0.03, 0.1, 0.3, 1.0, 3, 10, 30 and 100 µM. (**G**) shows that near maximal binding appeared when calcium ions reached 100 µM. (**H**) shows that no significant binding was detected when calcium ions were lower than 30 µM.

To examine whether S100P interacts with other cytoskeletal proteins, histidine-tagged S100P was immobilised on His-binding resin and used to capture proteins from cell lysates with blank beads as control. After extensive washings of the resin α, β-tubulins were detected in eluates, but only when 1 mM CaCl_2_ but not EGTA was originally present. Neither S100P nor tubulins were pulled down by blank beads control (Figure 2A). When α,β-tubulin was synthesised (Supplementary Table S1), immobilised on a chip and its binding affinity to S100P was determined using Surface Plasmon Resonance (SPR), the dissociation constant (mean ± SE) of the complex measured near equilibrium, *K*_d_ = 3.1 ± 0.3 × 10^−7^ M determined from multiple-cycle and 2.2 ± 1.6 × 10^−7^ M from single-cycle analyses (Figure 2B,C and Supplementary Table S2). The interaction could occur in both low, physiological (0.15 M) and high (0.5 M) NaCl buffers, equilibrium *K*_d_ = 61 ± 3.5 × 10^−7^ M (Figure 2D,E) and was dependent on calcium ions, which started to form the complex above 30 µM and saturated the reaction at 0.2 mM (Figure 2F–H).

### S100P forms complexes with tubulin in cells

**Figure 3.**
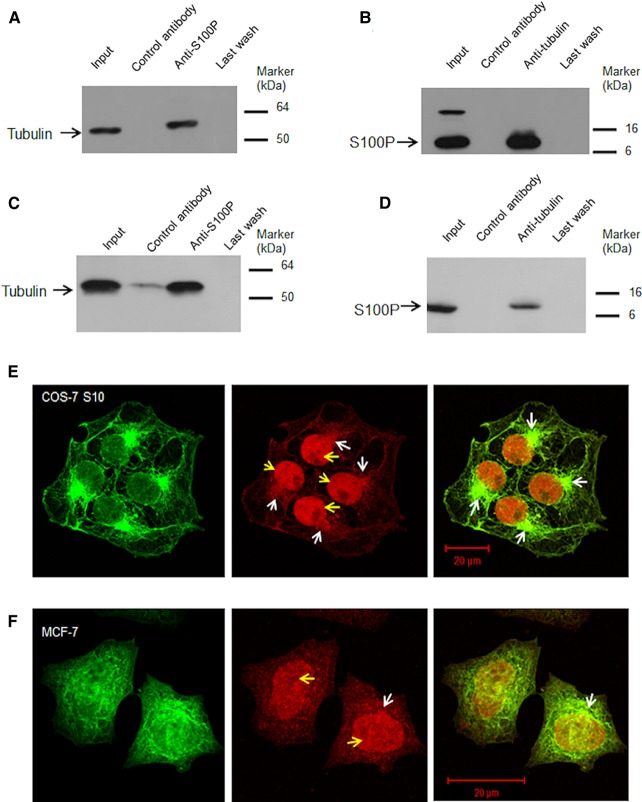
S100P binds to α,β-tubulins *in vivo*. (**A** and **B**), Co-immunoprecipitations in S100P transfected cells. (**A**) S100P-induced COS-7 S10 cell lysates were divided in three parts, one part was Western blotted directly for tubulin (Input), remaining parts were combined and immunoprecipitated with anti-S100P serum bound to beads. The eluate from the control antibody-bound beads (Control antibody), eluate from anti-S100P-bound beads (Anti-S100P) and last wash from the anti-S100P-bound beads (Last wash) were probed with anti-tubulin serum on Western blots. (**B**) Similar lysates were divided into three parts, one part was Western blotted (Input) for S100P and remaining parts were combined and immunoprecipitated with anti-tubulin-bound to beads. The eluate from control antibody-bound beads (control antibody), from anti-tubulin-bound beads (Anti-tubulin) and last wash (Last wash) were probed with anti-S100P serum. (**C** and **D**) Co-immunoprecipitations of native S100P. Co-immunoprecipitations of S100 and tubulin in MCF-7 cell lysates using (**C**) anti-S100P or (**D**) anti-β-tubulin as well as control antibody were undertaken. The eluates and last washes from the antibody-bound beads were subject to Western blotting using (**C**) anti-β-tubulin or (**D**) anti-S100P as described above (Methods). Control antibody in (**C**) showed a weak band, which may be due to a cross-reacting contaminant. However, this does not affect the conclusion that complex formation can occur between endogenous S100P and tubulin. (**E** and **F**) Typical confocal images of (**E**) COS-7 S10 cells induced for 24 h with 1 µg/ml doxycycline or (**F**) MCF-7 cells and both immunofluorescently stained with TRITC-labelled anti-S100P (red) and FITC-labelled anti-tubulin antibodies (green). Yellow arrows indicate the nuclei and white arrows indicate the mitotic organising centres (MTOC). Bars = 20 µm.

To demonstrate the existence of S100P-tubulin complexes in S100P-induced COS-7 S10 cells, potential complexes were immunoprecipitated with either anti-tubulin or anti-S100P and then probed with the reciprocal antibody. We found that the immunoprecipitates produced with anti-S100P also contained tubulin on Western blotting (Figure 3A) and *vice versa*, the immunoprecipitates with anti-tubulin contained S100P on Western blotting (Figure 3B). No bands were seen on Western blots of immunoprecipitates produced by control IgG serum (Figure 3A,B). A similar result was obtained using MCF-7 cells (Figure 3C,D) which expressed native S100P, tubulin and NMIIA (Figure 1C,D,F). These results show that S100P-tubulin complexes exist in lysed cells and suggest they may occur naturally in intact cells. However, using dually fluorescently labelled anti-S100P and anti-tubulin sera no clear association of S100P with peripheral MTs could be demonstrated except the co-localisation around MT-organising centres (MTOC) in S100P-induced COS-7 S10 cells (Figure 3E). In contrast, in poorly S100P-producing MCF-7 cells, the co-localisation could be seen only in a few cells and was not as obvious as in S10 (Figure 3F). These results suggest that S100P binds mainly to free α,β-tubulin subunits and MT-organising centres rather than to the peripheral MT filaments.

### Overexpression of S100P causes changes in MTs organisation

COS-7 cells were co-transfected with vectors expressing Cyan Fluorescent Protein (CFP) fused to End Binding Protein (EB3) (CFP-EB3) to highlight MT termini [17] and simultaneously with vectors expressing Yellow Fluorescent Protein (YFP) fused to S100P (YFP-S100P) to delineate S100P. We found that the cells expressing high levels of YFP-S100P showed a more dispersed distribution of cyan-labelled peripheral MTs than cells which expressed very low levels of S100P (Supplementary Figure S3A). YFP cDNA alone-transfected cells showed no effect on MTs (Supplementary Figure S3B). These data suggest that high levels of S100P may interfere with the distribution or formation of the MTs in living cells.

**Figure 4.**
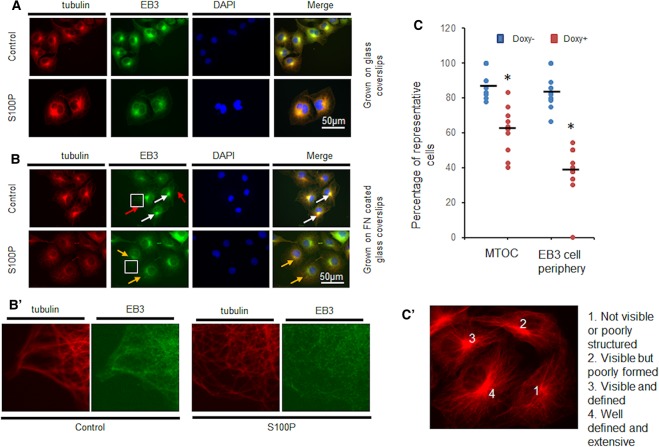
S100P expression leads to MTOC disruption and changes in EB3 localisation at the cell periphery in COS-7 cells. COS-7 S10 cells were induced for 48 h with 1 µg/ml doxycycline (S100P) or left untouched (Control) prior to seeding on (**A**) glass or (**B**) fibronectin (FN)-coated glass coverslips. After a further 48 h incubation in the same but fresh media, cells were dually immunofluorescently stained with TRITC-labelled antibodies to tubulin and FITC-labelled antibodies to End Binding Protein 3 (EB3). Nuclei were counterstained blue with DAPI. Typical images are presented. White arrows indicate the well-defined mitotic organising centres (dMTOC), red arrows indicate the clear EB3 bundles at cellular periphery. Yellow arrows indicate the MTOC that are not well-defined by EB3 staining. Bars = 50 µm. Images in **B′** correspond to the enlarged regions of the highlighted cells. (**C**) The number of well-defined mitotic organising centres (dMTOC) or EB3 bundles at the cellular periphery were counted and the percentage of cells containing such structures are presented as dot plots with bars representing means. *Student's *t*-test, *P* < 0.001 when S100P expressing cells (Doxy+) were compared with controls (Doxy−). Images in **C′** correspond to the quantification of how formed MTOC were quantified with cells showing (1) not visible or poorly structured organisation; (2) visible but poorly formed; (3) visable and defined; and well defined and extensive regions (Methods). Both MTOC organisation and EB3 localisation at the cell periphery were disrupted when S100P is expressed.

To determine whether altering S100P levels would lead to changes in MT-dependent organisation, S100P-inducible S10 cells were treated with 1 µg/ml doxycycline and seeded on coverslips with or without fibronectin, fixed and immunofluorescently stained red for tubulin and green for EB3 using TRITC and FITC labels, respectively. Some clear disruption could be seen in MTOC and in EB3 localisation at the cell periphery when S100P was expressed (Figure 4A,B) as well as in focused peripheral regions (panel B′). These changes were further exacerbated when cells were grown on fibronectin-coated coverslips where their adhesion and spreading was enhanced (Figure 4B). Whilst the overall structure of the MTOC was not compromised, there was much less distinct localisation, resulting in a reduction by a significant 31% in the percentage of well-defined (Methods) MTOCs per cell (Figure 4C). Equally, there was a significant reduction in 52% in the percentage of cells with bundles of EB3 at the cell periphery when S100P expression had been induced (Figure 4C).

### S100P slows down tubulin polymerisation in living cells

**Figure 5.**
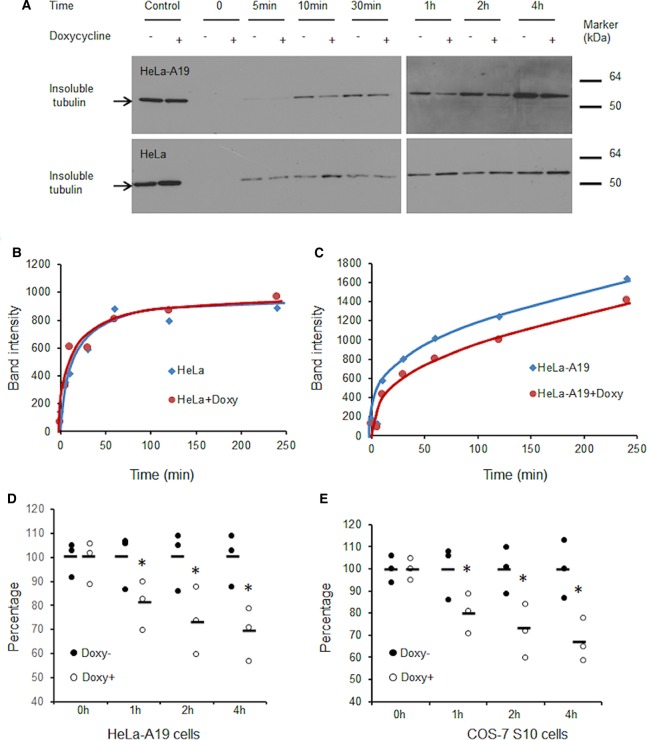
S100P slows down tubulin polymerisation after nocodazole treatment. HeLa, S100P-inducible HeLa (HeLa-A19) cells, COS-7 and S100P-inducible COS-7 (COS-7 S10) cells were synchronised by nocodazole for 24 h and released for 4 h to allow completion of cell division, then treated with nocodazole for 4 h to dissociate MTs, all without (−) or with (+) 1 µg/ml doxycycline (+Doxy). After the second release, the Triton-X100-insoluble tubulins were detected at subsequent times using Western blotting. Typical Western blots for (**A**) HeLa and HeLa-A3 and corresponding quantifications for (**B**) HeLa and (**C**) HeLa-A19 cells are presented. (**D** and **E**) Comparisons of the relative numbers of MTs at different times after release from nocodazole in the absence (Doxy−, set at 100%) or presence of 1 µg/ml doxycycline (Doxy+) in (**D**) HeLa-A19 or **E**, COS-7 S10 cells are presented as dot plots with bars representing means.*Student's *t*-test, *P* < 0.05 when compared to that at the same time but without doxycycline.

HeLa, HeLa-A19, COS-7 and COS-7 S10 cells were cultured without or with 1 µg/ml doxycycline and treated with 33 µM nocodazole for 24 h to synchronise the cells. The nocodazole was then washed away and the synchronised cells were cultured in normal medium for 4 h to complete cell division. The nocodazole was added again to dissociate all the MTs. After this time, nocodazole was removed to allow subsequent polymerisation of tubulin in the interphase of the cell cycle (Materials and Methods). The Triton-X100-insoluble tubulins (representing the newly formed MTs) were then detected at successive time points by Western blotting (Figure 5A) and their band intensities plotted against time after removal of nocodazole (Figure 5B,C). We found that tubulin polymerisation in S100P-induced cells was significantly slower (Student's *t*-test *P* < 0.05) by ∼30% after 4 h than that without S100P induction, in both S100P-inducible HeLa-A19 (31% reduced) and COS-7 S10 cells (32% reduced) (Figure 5D,E). In contrast, when HeLa or COS-7 parental cells were treated without or with 1 µg/ml doxycycline, the rates of tubulin polymerisation showed no significant differences (Figure 5B). These data indicate that S100P overexpression can slow down and change the dynamics of tubulin polymerisation in both NMIIA-containing and non-containing cells.

### Stabilising or disrupting MTs suppressed S100P-enhanced cell migration

**Figure 6.**
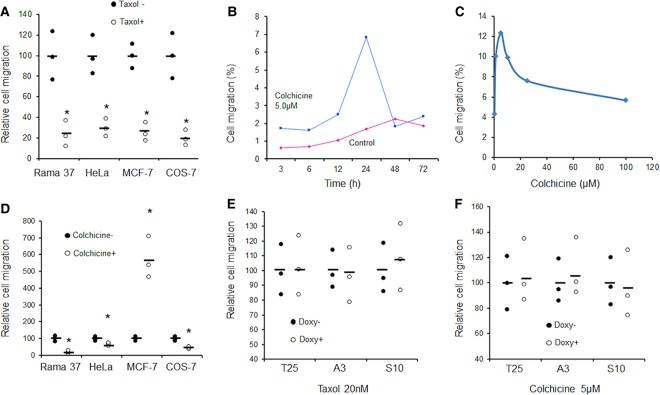
Effect of MT regulators on S100P-enhanced cell migration. (**A**) Dot plots of relative cell migration of different cell types in the absence (Taxol−, set at 100%) or presence of 20 nM taxol (Taxol+) over 24 h are presented with bars representing means. *Student's *t*-test, *P* < 0.01. (**B**) Time course of number of migrated MCF-7 cells relative to their input number expressed as a percentage in the absence (Control) or presence of 5 µM colchicine. The drop in migrated cells after 48 h is probably due to cell death. (**C**) Dose response of percentage changes in cell migration of MCF-7 cells to exposure to different concentrations of colchicine for 24 h. (**D**) Dot plots with bars representing means of relative cell migration over 24 h of different cell types are presented in the absence (Colchicine−, set at 100%) or presence of 5 µM colchicine (Colchicine+). *Student's *t*-test, *P* < 0.01. (**E** and **F**) Dot plots of relative cell migration of S100P-inducible cell lines: clone of Rama 37 T25 (T25), HeLa-A3 (A3), COS-7 S10 (S10) in the presence of (**E**) 20 nM taxol or (**F**) 5 µM colchicine without (Doxy−, set at 100%) or with 1 µg/ml doxycycline (Doxy+) over 24 h are presented with bars representing means. Student's *t*-test, *P* > 0.05 for all paired comparisons.

It has been reported that stabilising MTs by taxol significantly reduced cell migration in almost all cell types tested [17], whilst dissociation of MTs, by agents such as colchicine or nocodazole, had different effects on cell migration in different types of cells. It was reported that complete disruption of MTs in CHO and HeLa cells retained normal migration rates, but the resultant movement became more random [38] and reduced overall travel distance [39]. In Dicytostelial cells, nocodazole-disrupted MTs could significantly increase cell migration and shape changes, but reduced chemotaxis [40]. In our studies, we found that taxol significantly reduced cell migration in a dose-dependent manner (Supplementary Figure S4) and that 20 nM reduced migration by 70–75% in rat Rama 37, human HeLa, human MCF-7 and monkey COS-7 cell lines (Figure 6A). In contrast, colchicine was able to increase dramatically cell migration in a time and dose-dependent manner in MCF-7 cells only (Figure 6B,C), but significantly reduced migration in Rama 37, HeLa and COS-7 cells by 30–70% (Figure 6D). S100P-enhanced cell migration in Rama 37-T25, HeLa-A3 (both 3–4 folds) or COS-7 S10 cells (2–3 folds) was suppressed by either taxol (Figure 6E) or colchicine (Figure 6F). These data support the argument that dynamic changes of MTs are critical for cell migration and down-regulation of tubulin polymerisation could be a major mechanism of S100P-enhanced cell migration.

### Identify peptides which specifically interrupt S100P-tubulin interaction

To confirm that S100P-tubulin interaction is a major cause of S100P-enhanced cell migration, a specific disruptor of this interaction is required. To achieve this goal, we first mapped the binding sites of S100P on α, β-tubulin using a gel overlay assay with intact and half molecule tubulin fragments. However, S100P interacted with both α and β-tubulin subunits and with all four half molecules, suggesting the involvement of multiple binding sites (Supplementary Figure S5). Therefore a proteomic approach was utilised. Trypsin-digested α or β-tubulin peptides were applied to a S100P-conjugated Sepharose 4B column. After extensive washings, the bound peptides were eluted and characterised using mass spectrometry. The overlapping peptides were identified on the protein sequences of α, β-tubulin (Supplementary Figures S6, S7), presumably at potential binding sites. Based on published crystal structure of α, β-tubulin heterodimer, the overlapping peptides on its surface were selected, including two peptides from α-tubulin, one from its N terminus (starting with DLV), one from its C terminus (VNA), and two peptides from β-tubulin, one from the middle (DIC) and one from the C-terminal middle (LRF) (Supplementary Figures S6, S7). Another one was chosen that was identified by only a single nonoverlapping peptide IMN from β tubulin as a potential control. The identities of the selected peptides synthesised are shown in Supplementary Table S3.

**Figure 7.**
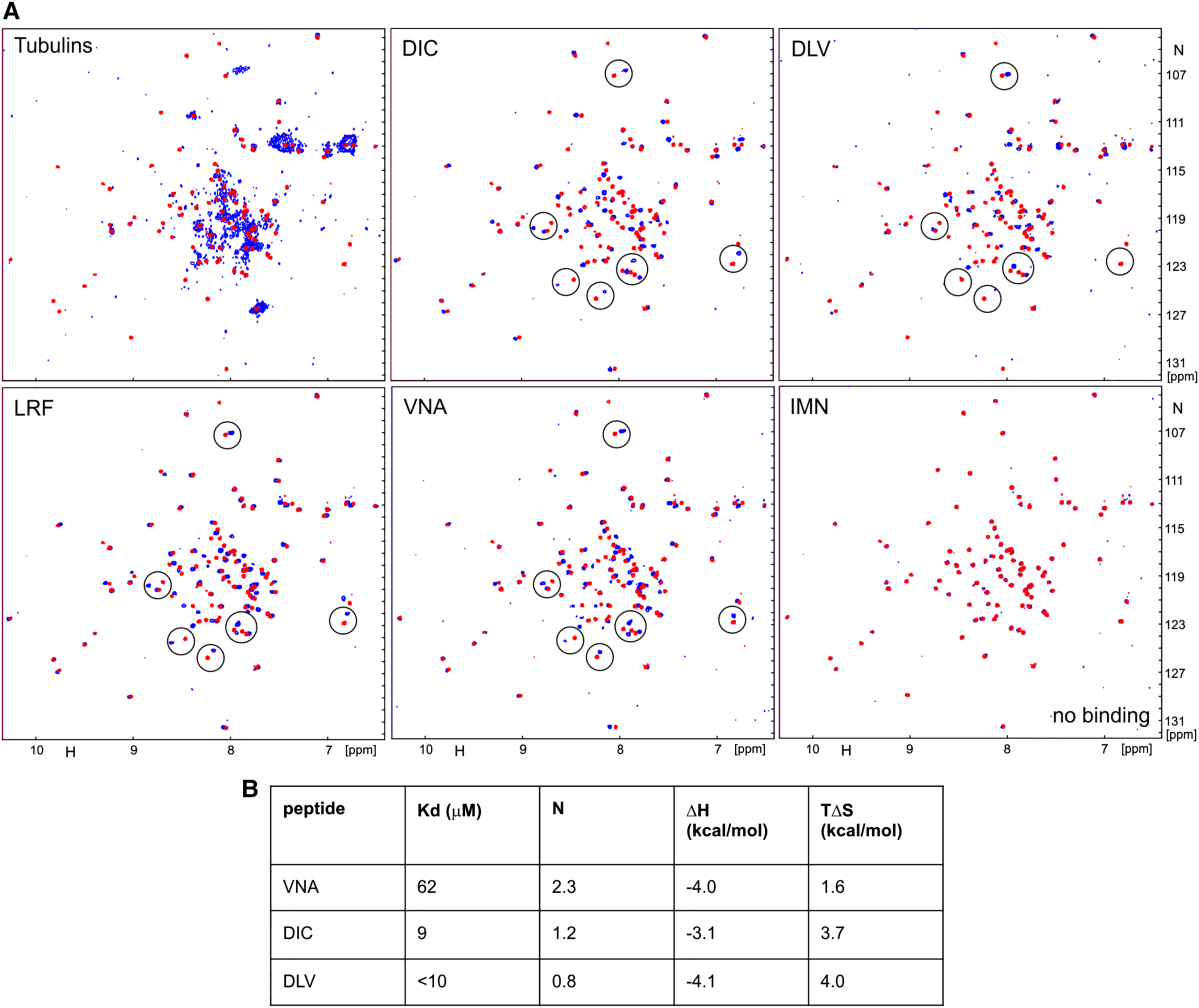
α, β-tubulin peptides bind to S100P. (**A**) Superposition of HSQC spectra of 0.1 mM ^15^N-labelled S100P in the free solution (red) and in the presence of equimolar concentration of α, β-tubulins (Tubulins) or different tubulin peptides (blue), as indicated on each panel. Examples of similar changes in HSQC spectra of peptides upon binding are encircled. (**B**) Thermodynamic parameters of the three best binding peptides from NMR data measured by isothermal titration calorimetry (ITC) calculated from the best-fit curves: dissociation constant (*K*_d_), molar ratio (*N*), enthalpy change (Δ*H*) and entropy change (Δ*S*) per mole.

The binding of the synthesised peptides to S100P was determined using NMR and ITC. Addition of IMN peptide to S100P had no effect on the NMR spectra of the protein, suggesting lack of interaction (Figure 7A). Four other peptides (DIC, DLV LRF and VNA) caused significant chemical shift changes in the HSQC spectra of ^15^N-labelled S100P, with similar signals affected (Figure 7A). This result supports the idea that these peptides interact with the same region(s) of S100P. Two of the peptides, DIC and VNA, were sufficiently soluble to measure their binding parameters by ITC (Figure 7B). Interaction with VNA is relatively weak, with *K*_d_ = 62 µM, but significantly stronger for DIC (*K*_d_ = 9 μM). The unexpectedly high stoichiometry of ∼2 (peptide:protein) for VNA may be due to some peptide aggregation, which is also suggested by the reduction in the intensity of its signals in the HSQC spectrum. HSQC signals were also weak for the DLV peptide, solutions of which were visibly cloudy even at low concentrations of 0.1 mM, clearly demonstrating peptide aggregation (Figure 7A). Thus low solubility of this peptide prevented accurate measurement of its affinity to S100P by ITC. However, the similarity of the observed chemical shift changes for DIC and DLV peptides suggested binding constant for DLV was similar to that measured for DIC < 10 μM. Overall, the *in vitro* experiments validated the interactions of S100P with four different regions of tubulin with low micromolar affinities; the affinity for the intact tubulin is expected to be higher through synergy between the different binding sites.

### Peptides that interrupt S100P-tubulin interaction significantly reduce S100P-enhanced cell migration

**Figure 8.**
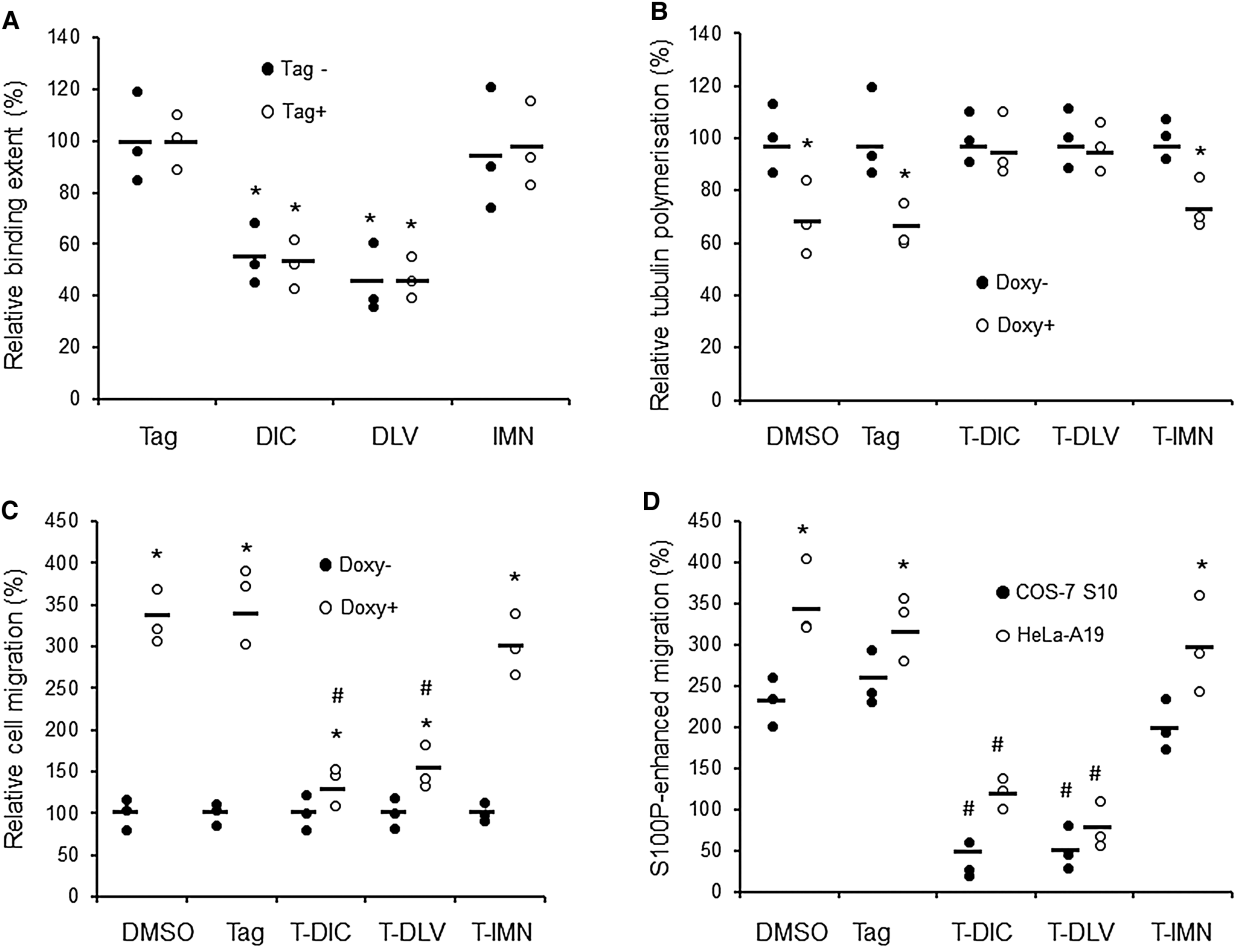
Effects of selected tubulin peptides on S100P binding, tubulin polymerisation and cell migration. (**A**) Surface Plasmon Resonance (SPR) assay to measure the effects of 20 µM selected peptides on the extent of binding of 1 µM S100P to a α,β-tubulin-coated chip. The peptides included DIC, DLV, and IMN without or with the Tag for cell entry, as well as the 10 amino acid Tag alone. *ANOVA test, *P* < 0.05 when compared to S100P alone set at 100%. (**B**) Effects of 20 µM tagged peptides (T-DIC, T-DLV, and T-IMN with Tag alone and solvent DMSO as controls) on S100P reduction in tubulin polymerisation after 4 h in 1 µg/ml doxycycline-induced COS-7 S10 cells. *Student's *t*-test; *P* < 0.05 when compare Doxy+ (with S100P induction) to Doxy− (without S100P induction, set at 100%). (**C**) Effects of 20 µM tagged peptides on S100P-enhanced cell migration after 8 h in 1 µg/ml doxycycline− induced COS-7 S10 cells. *Student's *t*-test *P *< 0.05 when compare Doxy+ (with S100P induction) to Doxy− (without S100P induction, set at 100%), T-DIC; *P* = 0.046. # *P* < 0.01 when compared to Doxy+ DMSO and Tag controls, indicating the inhibition of S100P-enhanced migration by peptides. (**D**) Comparison of S100P-enhanced relative cell migration after 8 h for NMIIA minus COS-7 S10 cells or NMIIA containing HeLa-A19 cells in the absence or presence of 20 µM tagged peptide (without S100P induction, set at 100%). #ANOVA test, *P* < 0.01 when compared to S100P-enhanced migration with addition of DMSO, Tag alone or T-IMN, the negative control. *Student's *t*-test *P* < 0.05 when compared to COS-7 S10 cells. In **A**–**D** dot plots of percentages are shown with bars representing means.

To test if three of the above peptides could block the interaction of S100P to immobilised α,β-tubulin on a SPR chip, 20 µM of each peptide (the minimal concentration determined to have maximal inhibitory effect), and 1 µM S100P were applied to the chip. We found that DIC and DLV peptides significantly inhibited the extent of binding of S100P by over 50% (Figure 8A). In contrast, the control IMN peptide which did not encompass the major overlapping S100P binding sequences on β-tubulin nor bind to S100P in the NMR (Figure 7A) showed no such effect (Figure 8A). Peptides containing N-terminal tags of 10 amino acids which enabled them to gain access to cells [35] were also synthesised and tested (Supplementary Table S3). The tagged peptides (T-DIC, T-DLV, and T-IMN) inhibited to the same extent as their untagged counterparts, since no significant differences between tagged and untagged moieties were observed. The Tag of 10 amino acids alone showed no effect in the binding assay (Figure 8A). The effect of tagged peptides on the S100P-induced delay in tubulin polymerisation in S100P-inducible COS-7 cells was then examined. Twenty micromolar T-DIC and T-DLV, but not control T-IMN completely abolished the 30% inhibition of tubulin polymerisation caused by induction of S100P (Figure 8B). These results are consistent with the binding assays.

Although doxycycline-induction of S100P significantly increased cell migration with all of the tagged peptides at 20 µM, the extents were very different. The S100P-induced 3.4 or 3.5 fold increase in the presence of DMSO or Tag was significantly reduced to 1.4 or 1.5 fold with T-DIC or T-DLV, respectively, but not significantly with T-IMN peptide (Figure 8C). Thus in the presence of T-DIC or T-DLV, S100P-enhanced cell migration in both NMIIA minus COS-7 S10 and NMIIA plus HeLa-A19 cells was significantly reduced by ∼60–90% compared to that with DMSO or Tag alone controls, but there was no significant reduction with T-IMN peptide (Figure 8D). These data show that the effect of S100P on tubulin polymerisation is a major mechanism for S100P-enhanced cell migration in NMIIA-containing, as well as in NMIIA-depleted cells.

## Discussion

Movement away from the primary tumour is the critical first step in metastasis [41,42]. Previously we have demonstrated that both S100A4 and S100P are able to promote metastasis in syngeneic rat models for breast cancer and that their overexpression in human primary breast cancers is associated with poor patient prognosis [5,9,43] due to metastasis, at least to the brain [44,45] and the liver [46]. Moreover, high levels of S100A4 [12] and S100P [10] are able to increase cell migration, in part, by binding to the NMIIA component of the actomyosin cytoskeleton, so as to reduce levels of actomyosin filaments and focal adhesions to the cellular substrata. In this way, the cell adhesive force is reduced and the driving force of NMIIB is left relatively unopposed. These changes facilitate the detachment of cancer cells and promote their spread through the extracellular matrix [10]. In the current study, we report that overexpression of S100P in a cell line, COS-7 without any detectable NMIIA, can still enhance cell migration, but without a corresponding decrease in cell adhesion (Supplementary Figures S1, S2). This work is the first to establish a direct cause and effect relationship between S100P and cell migration that is independent of the actomyosin cytoskeleton and adhesive properties of cells. Furthermore, we show that this S100P-induced increase in cell migration occurs instead through the interaction of S100P with α,β-tubulin and its consequent reduction in tubulin polymerisation in these NMIIA-depleted COS-7 cells. That S100P and tubulin interact *in vivo* has been demonstrated by pulldown experiments from lysed cell extracts using antibodies to either component separately (Figure 3A,B) and by use of externally-added fluorescently-labelled antibodies to permeabilised cells (Figure 3E,F). To eliminate the possibility that complex formation occurs after cell lysis or indeed after permeablisation, co-localisation of endogenously fluorescently-labelled S100P and EB3-capped tubulin was also shown to occur in intact living cells (Supplementary Figure S3).

Cell migration is a complicated biological process involving many disparate cellular machines and regulatory systems [47]. The cytoskeleton is essential for cell migration and is composed of three major types of fibres, intermediate filaments, actomyosin filaments and MTs [48]. The intermediate filaments mainly provide mechanical strength to cells, whilst actomyosin filaments directly provide forces for cell migration [49]. The roles of MTs in cell migration are more complex. On the one hand, MTs are a major support to cell polarity and their assembly can provide force for directional movement [50]. On the other hand, MTs make a cell's shape more rigid and thereby reduce their motile efficiency. From our experiments, we find that S100P can interact with nonpolymerised α, β-tubulin in test tubes using three independent assays: binding of cell lysates to His-tagged S100P beads (Figure 2A), SPR binding to immobilised tubulin (Figure 2B–H) and gel overlay assay (Supplementary Figure S5). The fact that such complexes can occur in intact cells is demonstrated using immunoprecipitation of cell lysates (Figure 3A,B). All 3 assays exhibit the same requirement for Ca^2+^ ions before interaction is observed. Once the Ca^2+^ concentration drops to 30 µM no binding signal of S100P to tubulin is detectable in the SPR assay which saturates at 200 µM (Figure 2F–H). These concentrations are high, average Ca^2+^ levels are 100 nM or so in cells, although it is not unusual to see intracellular concentrations of 0.5 µM following signalling activation [51]. These concentrations may arise in distinct microdomains within the cytoplasm caused by opening of specific Ca^2+^ channels within the plasma membrane or endoplasmic reticulum [52–55]. These concentrations may still represent an apparent discrepancy in that many S100 proteins bind Ca^2+^ in the 10–50 µM range *in vitro*, well above the level observed *in vivo* [56]. However, when binding of Ca^2+^ to preformed S100A4/NMIIA fragments was studied using ITC, its *K*_d_ value was reduced by several orders of magnitude [57]. In addition to S100A4, S100B and several other S100 proteins bind Ca^2+^ ions relatively weakly in the absence of protein targets (*K*_d_ > 1 µM). In contrast, when they bind to their target proteins, Ca^2+^ binding can increase by as much as 200 to 400 fold [58]. Moreover, to observe binding of S100P to interferon β [59] or V domain of RAGE [60] using either SPR or ITC/tryptophan fluorescence, respectively, Ca^2+^ concentrations of 1 to 4 mM were employed. Thus it may be that the local intracellular concentrations of Ca^2+^ peak at higher values than those observed on average in the cytoplasm of activated cells and/or the requirement for Ca^2+^ ions is greatly reduced by the configuration of S100P-tubulin perhaps complexed with additional proteins/ions *in vivo* compared to that *in vitro*.

In our hands, the *K*_d_ of the interaction between S100P and tubulin from SPR analysis is 2–3 × 10^−7^ M, as calculated from estimates at or near equilibrium or 1–2 × 10^−7^ from a ratio of forward and backward rate constants [61]. Moreover, binding of S100P to tubulin is shown to occur throughout both α,β forms via multiple binding sites (Supplementary Figures S5–S7). Thus S100P interacts mainly with unpolymerised α,β-tubulin, presumably to prevent its assembly into MTs (Figure 5E) and thereby may change MTs dynamics. These results are also consistent with those found previously for the binding of S100A1 or S100B to tubulin and their reduction in assembly into MTs in brain cells [24,25,62,63]. Using either living colours (Supplementary Figure S3) or antibody labelling (Figures 3 and 4) to monitor MTs, we find that in both cases overexpression of S100P causes the MT filaments to be less distinctive. In particular, the EB3-capped MTs at the cellular peripheries are significantly reduced by ∼52% and there is a 31% loss in MTOCs per cell (Figure 4); results which are consistent with the near steady-state reduction in 33% in biochemical assembly of MTs (Figure 5E). Localisation of S100P is mainly nuclear and perinuclear (Figure 3E). Distinctive co-localisation of S100P with α, β tubulins mainly appears in MTOCs (Figure 3E) in COS-7 cells. All these results suggest that overexpression of S100P can affect the dynamics of MTs within COS-7 cells. This conclusion is not due to lack of NMIIA in COS-7 cells, since a virtually identical reduction in rate of polymerisation of tubulin is observed in NMIIA-containing, S100P-inducible HeLa cells (Figure 5D,E).

The chemicals taxol and colchicine have much stronger effects on tubulin polymerisation than those of S100P (Figure 6). Taxol, a stabiliser of MTs [64], significantly reduces cell migration in all cell lines tested (Rama 37, HeLa, MCF-7 and COS-7), even at a very low concentration of 20 nM (Figure 6A). This concentration completely prevents the S100P-induced stimulation of migration in the NMIIA-depleted (COS-7), as well as in the NMIIA-containing (Rama 37, HeLa, MCF-7) cells (Figure 6A). In contrast, colchicine, an MT disrupter [19–21], enhances cell migration in a time (Figure 6B) and concentration (1–10 µM) dependent manner in MCF-7 cells (Figure 6C), although longer times of incubation (>24 h) or higher concentrations (>10 µM) abolishes this effect (Figure 6B,C), However the same concentration of 5 μM which stimulates cell migration in MCF-7 cells inhibits migration of Rama 37, HeLa and COS-7 cells (Figure 6D). These observations strongly suggest that excessive MT formation or complete disruption definitely inhibits cell migration and overrides any stimulation produced by induction of S100P in our experiments (Figure 6E,F). In contrast results for subsaturating concentrations of colchicine, at least for MCF-7 cells (Figure 6B,C), suggest that in the case of the NMIIA-depleted cells, S100P and colchicine may be stimulating the same pathway for cell migration. However, S100P-enhanced cell migration appears to be caused not simply by blocking MTs assembly, but more likely by changing MTs dynamics. Moreover, there are other examples of proteins such as CAP-Gly domain-containing proteins P120-catenin [65] and certain pathways such as PI3 kinase-Akt [66] that regulate the dynamics of MT and can affect cell migration. For example overexpression of P120-catenin can stabilise the MTs and reduce cell migration, whilst knocking-down P120-catenin can destabilise the MTs and increase cell migration [65].

Despite the many reports which have shown that disruption of MTs can impair cell migration, particularly directional migration, cells can still retain the ability of persistent motility without MTs [67], presumably using other components of the cell's cytoskeleton [47]. Well-established MTs may increase the rigidity of cells as well as their polarity and play a constraining role to limit a cell's ability of moving and changing direction [68]. Disrupting MTs using nocodazole can dramatically enhance the non-directional (random) movements of cells [40]. However, unlike the scratch wound-healing assay which relies on directional migration of cells, the transwell migration assay used here is a reliable method to measure the cell's non-directional migration in space, although a nutrient gradient of 2 to 10% (v/v) FCS is applied across the membrane separating the chambers [69,70].

**Figure 9.**
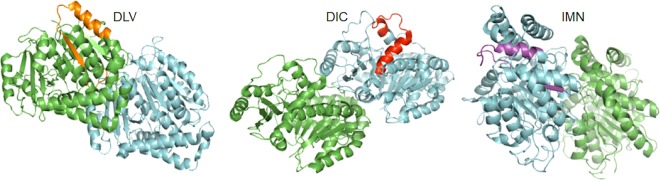
Position of the peptides in the structure of α,β-tubulin dimer. The locations of peptides DLV, DIC and IMN in α,β-tubulin dimer crystal structure from PDB database (Ref 1JFF) (green: α-tubulin, cyan: β-tubulin) are shown in different colours. DLV and DIC are on the surfaces which are involved in tubulin polymerisation. IMN is partially buried.

To prove that the effect of S100P on tubulin is the major mechanism of S100P-enhanced cell migration in NMIIA-depleted COS-7 cells, we have used the two strongest S100P-binding peptides tested in ITC, the 30mer starting with DLV from α-tubulin and the 26mer starting with DIC from β-tubulin, as well as a very weak or nonbinder as a control, the 30mer starting with IMN from β-tubulin (Supplementary Table S3; Figure 7). The location of these peptides in the α,β-dimer crystal structure suggest that DLV and DIC are on surfaces which are involved in tubulin polymerisation whilst IMN is partially burried (Figure 9) probably weakening IMN's ability to bind to S100P. They were all tagged with a S100P-binding-inert (Figure 8A) 10 amino acid peptide (T) to enable them to enter cells [35]. Since the two peptides, T-DIC and T-DLV which cause a significant 50% reduction in extent of binding of S100P to tubulin (Figure 8A), virtually abolish both the S100P-induced decrease in tubulin polymerisation (Figure 8B) and the S100P-induced increase in cell migration (Figure 8C,D), the binding of S100P to α, β-tubulin would appear to be an obligatory step in the overall process. Moreover, since there is no change in either tubulin polymerisation or cell migration produced by these two tagged peptides in uninduced COS-7 cells (Figure 8B,C), the two binding sites on intact α, β tubulin represented by the DIC and DLV-containing peptides are probably uniquely recognised by S100P and perhaps similar S100 proteins.

In the case of NMIIA-containing HeLa cells, the two inhibitory peptides DIC and DLV still significantly blocked the S100P-induced increase in cell migration, but only by 49% and 54%, respectively. This result suggests that, although part of the S100P-induced stimulation of cell migration in HeLa cells is caused by S100P binding to tubulin, the remainder is caused by another mechanism, in this case probably S100P binding to NMIIA [10]. These results are consistent with the fact that the tagged NMIIA peptide which inhibits the binding of S100P to NMIIA, reduces S100P's stimulation of migration by ∼50% [10], leaving ∼50% of stimulation unassigned. It is suggested that this unassigned stimulation is due to S100P interacting with tubulin and that S100P can bind to both NMIIA [36] and α, β-tubulins in cells containing NMIIA to propagate two independent pathways that stimulate cell migration. Since coordination of MTs and actomyosin filaments is important in cell migration [48,71], S100 proteins like S100P or S100A4 may undertake this role in promoting cell migration, particularly in the neoplastic cell. Therefore, one possible reason for the widespread occurrence of certain S100 proteins in metastatic cancers [72–74] may be due to the promiscuous nature of their interactions with proteins linked to stimulating cell migration and invasion, the first major step in the metastatic process.

## Open Access

Open access for this article was enabled by the participation of University of Liverpool in an all-inclusive *Read & Publish* pilot with Portland Press and the Biochemical Society under a transformative agreement with JISC.

## Abbreviations

CB: cytoskeletal buffer
CFP: cyan fluorescent protein
EB3: end binding protein 3
ITC: isothermal calorimetry
mAb: monoclonal antibody
MES: 2-morpholinoethane sulphonic acid
NM: nonmuscle myosin
NMIIA: nomuscle myosin IIA
SPR: surface plasmon resonance
TCEP: tris-carboxyethyl-phosphine
T-DIC: tagged for cell entry followed by first three N-terminal amino acids of the peptide
YFP: yellow fluorescent protein
