# Supplementary material for: Direct interaction of metastasis-inducing S100P protein with tubulin causes enhanced cell migration without changes in cell adhesion
Source: Biochem J. 2020 Mar 27;477(6):1159–78. doi: 10.1042/BCJ20190644 (PMC7108782; doi:10.1042/BCJ20190644)
Supplement: Supplementary Tables S1-S3 and Figures S1-S7 [file BCJ-477-1159-s1.pdf]

Supplementary Tables

Supplementary Table S1: Primers used for recombinant protein production

Peptides	5' primer	3' primer
α tubulin	CGTATCCACTCATATGCGTGAGTGCATCTCCATCC	CTACTGGATCCTTAGTATTCCTCTCCTTCTTCCTC
α tubulin N-terminus(αN)	CGTATCCACTCATATGCGTGAGTGCATCTCCATCC	CTACTGGATCCTTAATATGTGGCCAGAGGGAAGTG
α tubulin C-terminus(αC)	CGTATCCACTCATATGCCTGCATCTCTGCTGAGAAAG	CTACTGGATCCTTAGTATTCCTCTCCTTCTTCCTC
β tubulin N-terminus(βN)	GATGCGTAGCATATGCGTGAAATTGTCCATATTCAG	TAGATGGATCCTTAGATGTCATAGAGGGCCTCATTG
β tubulin C-terminus(βC)	GATGCGTAGCATATGTTCCGTACCCTGAAGCTGACG	GATGCGTAGCATATGCGTGAAATTGTCCATATTCAG

Supplementary Table S2: Summary of SPR binding of S100P to αβ-tubulin

Buffer (procedure)	Equilibrium dissociation constant Kd x10^7	
	Kinetic[a]	Equilibrium[b]
150mM NaCl, 0.5mM $CaCl_2$ (single cycle)	1.2±0.12	2.2±1.6
150mM NaCl, 0.5mM $CaCl_2$ (multiple cycles)	2.1±0.9	3.1±0.3
300M NaCl, 0.5mM $CaCl_2$ (multiple cycles)	8.9±0.9	11±1.6
500M NaCl, 0.5mM $CaCl_2$ (multiple cycles)	83±7	61±3.5
150M NaCl, EDTA 5mM (multiple cycles)	1L[c]	1L[c]

[a] Mean equilibrium dissociation constant Kd calculated from the association and dissociation rate constants using SPR (Materials and Methods) ± SE from 3 separate experiments.

[b] Mean Kd calculated from extent of binding at or near equilibrium ±SE from 3 separate experiments.

[c] 1L incalculably large.

Supplementary Table S3: Identity of tubulin-related peptides synthesised

Fragment[a], Region of tubulin[b].	Peptide identifier[c]	Sequence synthesized	Size (AA)
α-N, 116-145	1	DLVLDRIRKLADQCTGLQGFLVFH-SFGGGT	30
α-C, 328-357[d]	2	VNAAIATIKTKRTIQFVDWCPTGFK-VGINY	30
β-N, 163-190[d]	3	IMNTFSVVPSPKVSDTVVEPYNATL-SVH	30
β-N, 209-234	4	DICFRTLKLTTPTYGDLNHLVSATMS	26
β-C, 240-264	5	LRFPGQLNADLRKLAVNMVPFPRLH	25
Non muscle myosin IIA peptide[e]		ASTRLKQLKRQLEEAEEEAQRANAS-RKLQRELEDATETADAMNREVSSL-KNKLRRGDLPFVVPRRMARKG	71
Tag		RRRQRRKKRG	10
Tagged DLV	1	RRRQRRKKRGDLVLDRIRKLADQCT-GLQGFLVFHSFGGGT	40
Tagged DIC	4	RRRQRRKKRGDICFRTLKLTTPTYGD-LNHLVSATMS	36
Tagged IMN	3	RRRQRRKKRGIMNTFSVVPSPKVSDT-VVEPYNATLSVH	40

[a] Half molecule fragments containing N or C terminal of α or β-tubulin

[b] Region defined by amino acid residues in α or β-tubulin

[c] Arbitrary peptide identifying number (Supplementary Figs S11, S12)

[d] Substitute one AA in peptide from original sequence

[e] Defined in ref 10

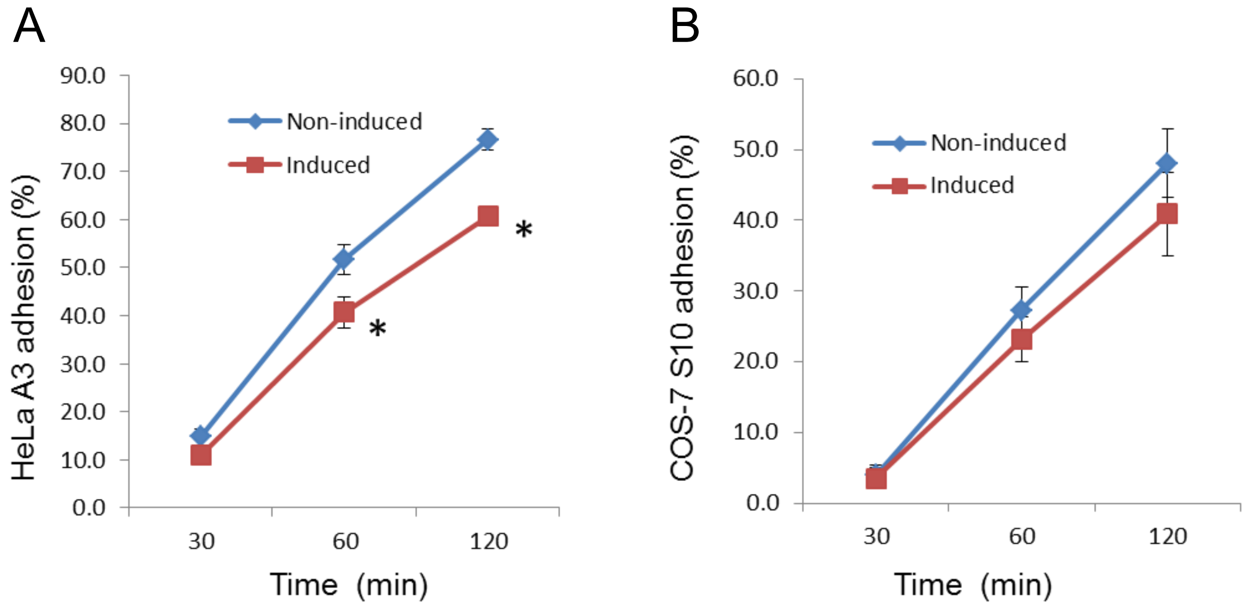

Supplementary Figure S1. Rate of adhesion assay

Percent cells remaining that have adhered to tissue culture wells at different times after initial seeding are shown (Materials and Methods). (**A**) Control uninduced HeLa A3 cells and doxycycline-induced HeLa A3 cells. Differences at 30 min Student's t = 3.6, P = 0.022; 1 hour t= 4.3, P= 0.013; 2 hour t = 9.3, P = 0.0007, n =3. (**B**) Control uninduced COS-7 S10 cells and doxycycline-induced COS-7 S10 cells. Differences at 30 min t = 0.4, P = 0.69; 1 hour t = 1.6, P = 0.19; 2 hour t = 1.6, P = 0.18, n = 3. Means +/- SD of each experiment (n=3) are shown. Asterisk (*) indicates significantly different between uninduced control and S100P-induced cells (Student's t-test, P < 0.05).

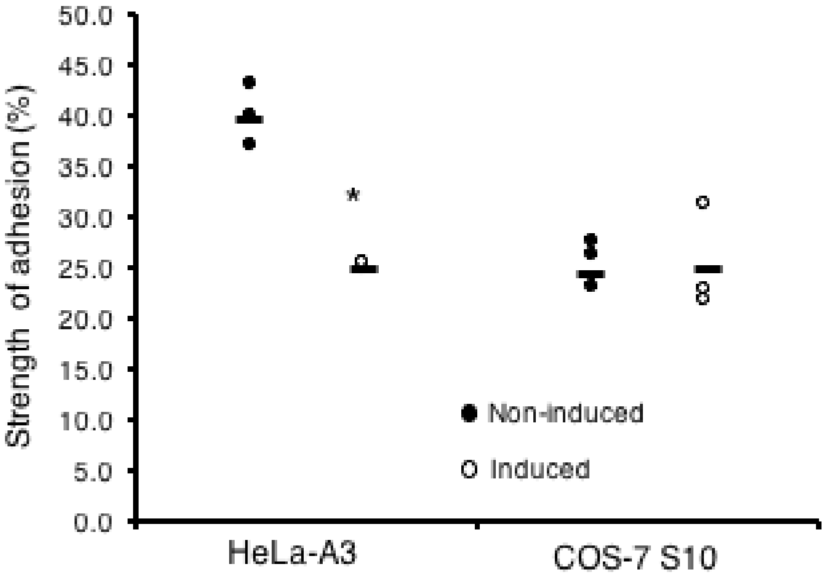

Supplementary Figure S2. Strength of adhesion assay

Cells (80% confluent) were predigested with dilute 0.0125% (w/v) trypsin in versene for 5 min to remove weakly bound cells. The remaining bound cells were removed and counted using standard procedures. Values are expressed as a percentage of the original number of cells seeded. When control uninduced HeLa A3 cells and doxycycline-induced HeLa A3 cells were compared, there was a significant 1.58 fold decrease in the strength of adhesion upon induction of S100P (Student's $t = 6.7$, $P = 0.022$, $n = 3$). When uninduced COS-7 S10 cells and doxycyclin-induced COS-7 S10 cells were compared there was no significant change in the strength of adhesion upon induction of S100P ($t = 0.6$, $P = 0.95$, $n = 3$). Asterisk (*) indicates significantly different between uninduced control and S100P-induced cells (Student's t-test $P < 0.05$).

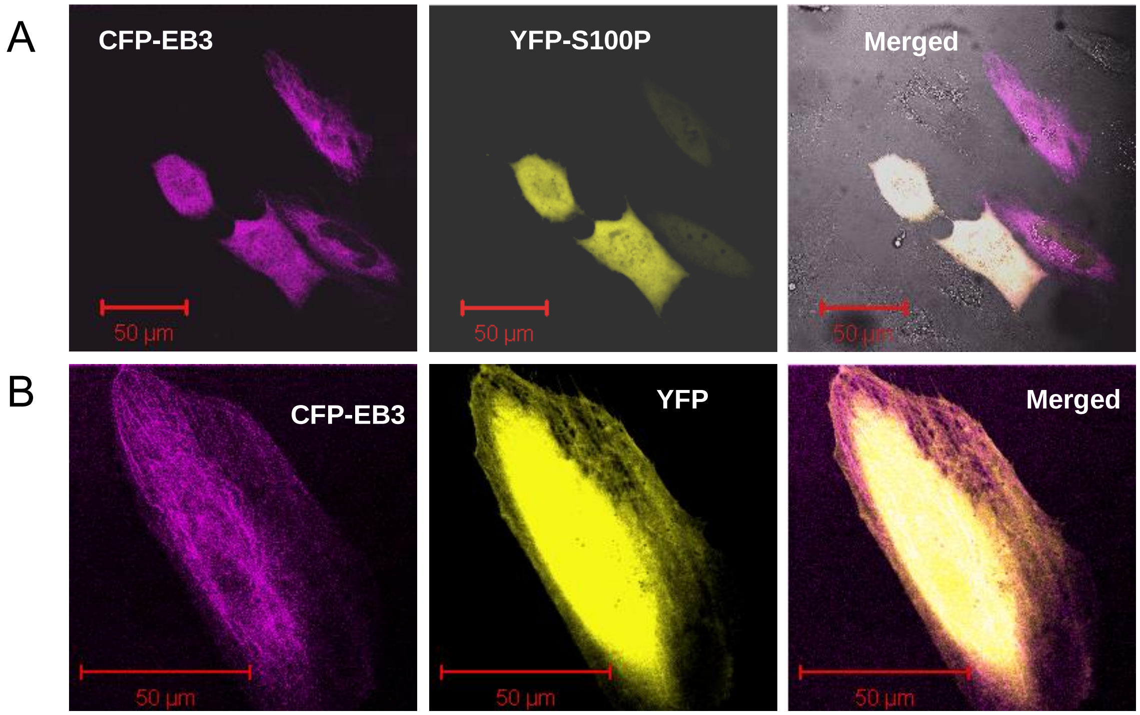

Supplementary Figure S3. Effect of S100P on the distribution of MTs in COS-7 cells. Upper panels (A): COS-7 cells were transiently co-transfected with expression vectors for Cyan Fluorescent Protein coupled to End Binding Protein 3 (CFP-EB3) and for Yellow Fluorescent Protein coupled to S100P (YFP-S100P) and photographed after 24h. **Lower panels (B):** COS-7 cells were transiently co-transfected with expression vectors for CFP-EB3 (Cyan) and for YFP alone (Yellow) as a control and photographed for the same time. Typical images are presented. Bars=50µm.

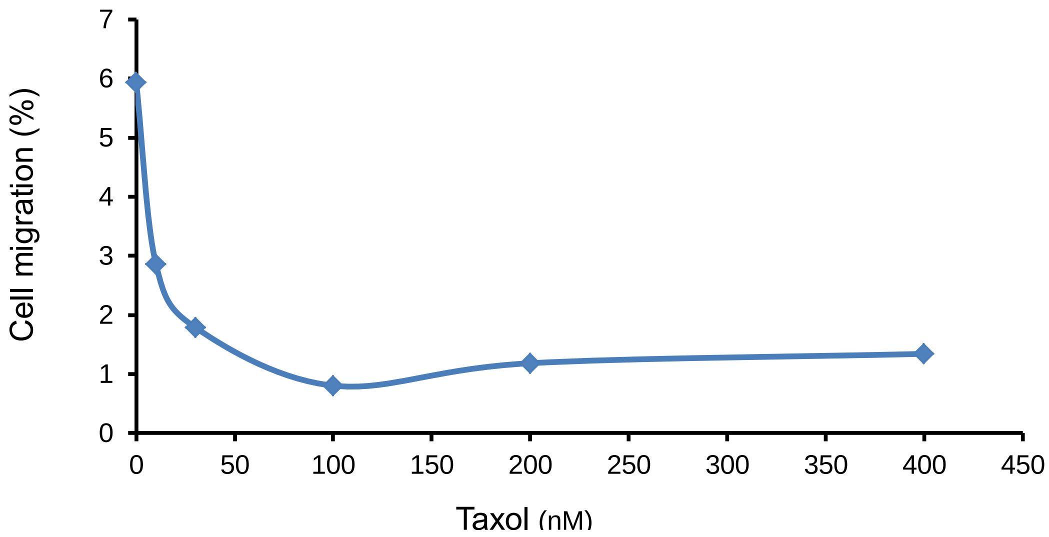

Supplementary Figure S4. Effect of different concentrations of taxol on cell migration of COS-7 cells.

The percentage of input cells which migrated after 24h is shown for different concentrations of taxol in nM.

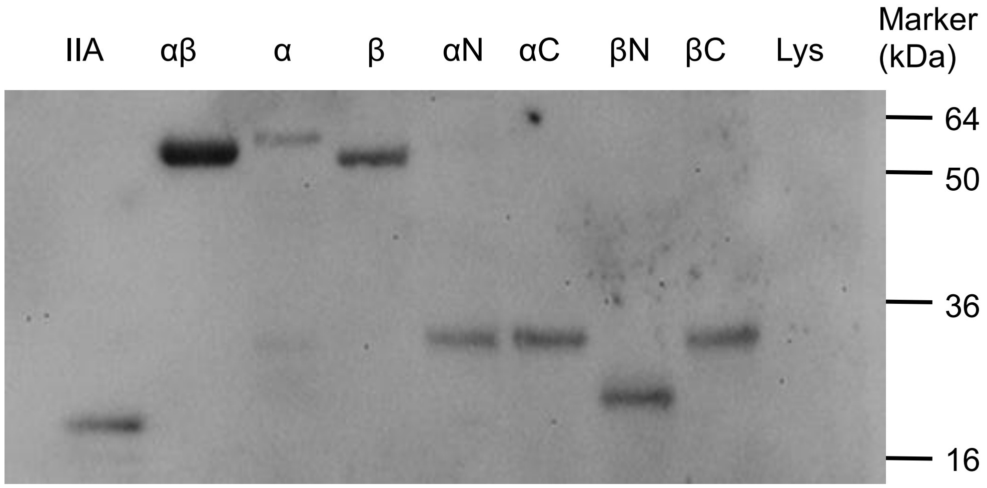

Supplementary Figure S5. Gel overlay assay

Equal molar concentrations of control lysozyme (Lys) as negative control, nonmuscle myosin peptide (IIA, as positive control; Supplementary Table S3; ref 10), αβ-tubulin dimer (αβ), His-α-tubulin (α), β-tubulin (β), α-tubulin N/C-terminus half molecules (αN, αC); β-tubulin N/C terminus half molecules (βN, βC) were subjected to SDS-PAGE on 10% (w/v) polyacrylamide gels, transferred onto PVDF membrane and incubated with 3 µg/ml S100P protein. The bound S100P was probed with anti-S100P serum and visualized with ECL (Methods).

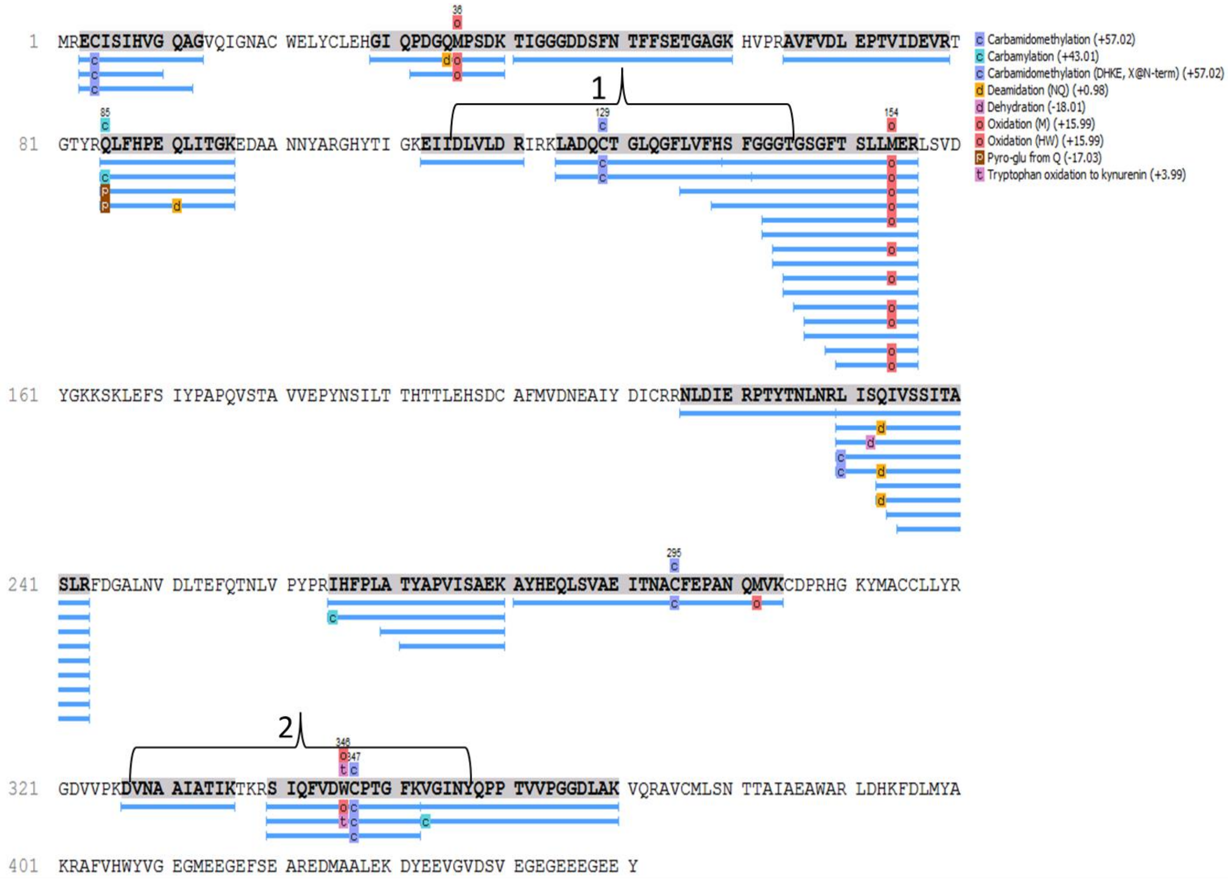

Supplementary Figure S6. Identification of α-tubulin peptides that bind to S100P

Tryptic digests of α-tubulin were applied to a S100P-conjugated Sepharose 4B column. After extensive washings, any bound peptides were eluted with 0.1M Glycine pH 2.5 and analyzed in a mass spectrometer (Dundee University Mass Spectrometry Service). The different peptides in blue were aligned qualitatively on the known human α-tubulin sequence using PEAK 7 software. Modifications introduced prior to or during the mass spectrometric analysis are also shown. The peptides labelled 1 and 2, selected for synthesis in Supplementary Table S3, are also shown superimposed on the amino acid sequence for α-tubulin.

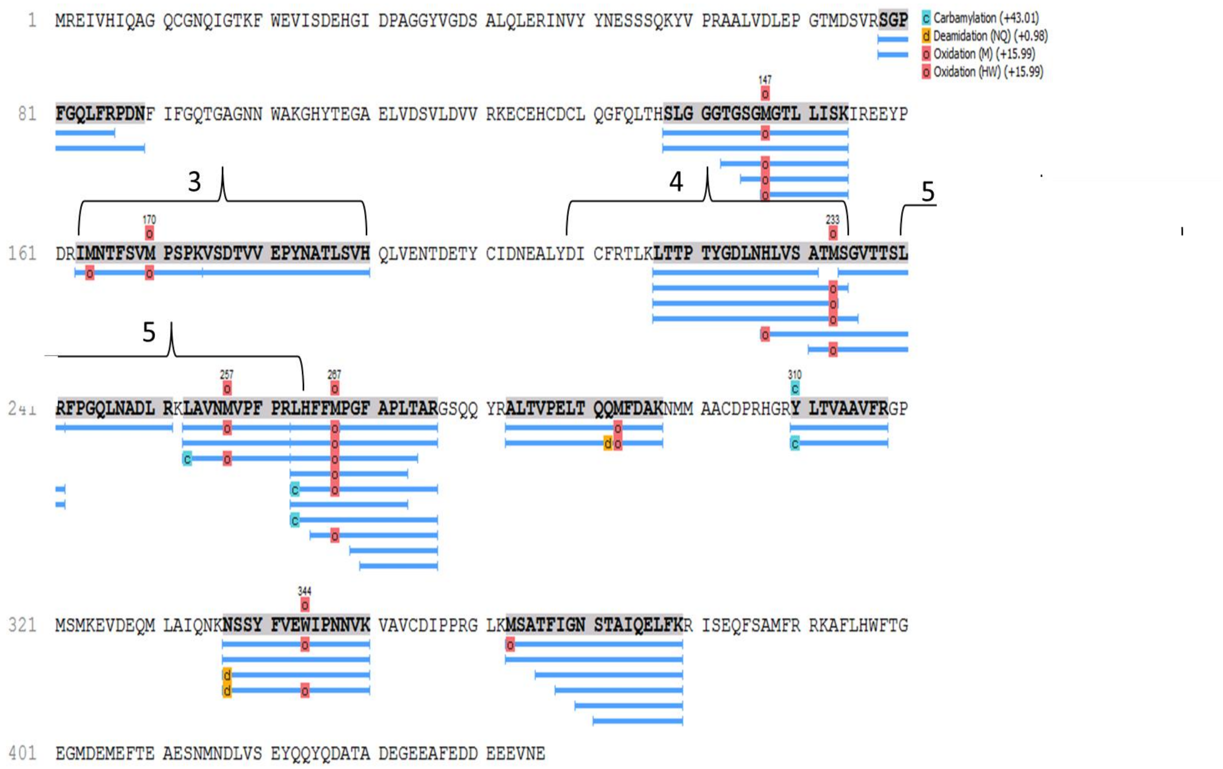

Supplementary Figure S7. Identification of β-tubulin peptides that bind to S100P

Similar to Figure S6, the alignments of the mass spectrometry-identified sequences of isolated β-tubulin tryptic peptides are shown in blue. These sequences were obtained from mass spectrometric analysis of tryptic digests of β-tubulin bound to S100P-conjugated Sepharose 4B beads using PEAK 7 software. The peptides labelled 3-5 selected for synthesis in Supplementary Table S3 are shown superimposed on the amino acid sequence for β-tubulin.
